# Post-marketing safety concerns with Tislelizumab: a disproportionality analysis of the FDA adverse event reporting system

**DOI:** 10.3389/fimmu.2025.1596842

**Published:** 2025-05-26

**Authors:** Chen Li, Yi Ding, Shanshan Cai, Bai Cheng Liu, Xiufeng Wang

**Affiliations:** ^1^ Clinical Discipline Construction Center, Graduate School of Shanxi Medical University, Taiyuan, China; ^2^ Department of Orthopedic Trauma, Zhuji People's Hospital of Zhejiang Province, Zhuji, China; ^3^ The Department of Pulmonary and Critical Care Medicine, The Second Hospital of Jilin University, Changchun, China; ^4^ Division of Biomedical and Life Sciences, Faculty of Health and Medicine, Lancaster University, Lancaster, United Kingdom; ^5^ Division of Urology, Shanxi Medical University Affiliated Lv liang Hospital, Lvliang, China

**Keywords:** tislelizumab, FAERS, adverse events, immune checkpoint inhibitors, irAEs

## Abstract

**Background:**

Tislelizumab is an anti-programmed cell death protein 1(anti-PD-1) monoclonal antibody, which was approved by the Food and Drug Administration(FDA) on March 14, 2024. However, clinical studies are often limited by small sample sizes, and thus a more comprehensive evaluation of the safety of Tislelizumab, particularly its immune-related adverse reactions, is urgently needed.

**Method:**

Disproportionality analysis was used in this study to assess the safety of Tislelizumab in clinical practice by analyzing all adverse event reports from the FDA Adverse Event Reporting System database, starting from the first quarter of 2024, where Tislelizumab was identified as the primary suspected drug. Two disproportionality analysis methods, reporting odds ratio (ROR) and Bayesian confidence propagation neural network (BCPNN), were utilized to investigate the adverse reactions related to Tislelizumab. Additionally, the Weibull distribution was employed to examine the time-dependent changes in the incidence of adverse events.

**Results:**

Consistent with the drug label, this study identified significant positive signals for adverse reactions, including myelosuppression, hepatic dysfunction, pruritus, rash, and exfoliative dermatitis. Notably, this study also identified several adverse reactions not documented in the drug label, including palmar-plantar erythrodysaesthesia syndrome, immune-mediated cystitis, and renal cysts. Adverse reactions associated with Tislelizumab generally manifested within the first month of treatment. In terms of immune-related adverse reactions, Tislelizumab demonstrated lower signal values compared to other immune checkpoint inhibitors.

**Conclusion:**

This study comprehensively reviews the safety profile of Tislelizumab, thereby providing clinicians with crucial safety information for prescribing this drug. Due to its relatively low risk of immune-related adverse events (irAEs), Tislelizumab may serve as a promising candidate for combination therapy with other immune checkpoint inhibitors (ICIs). Novel combination strategies involving Tislelizumab and other ICIs are anticipated to provide new therapeutic opportunities for patients experiencing irAEs.

## 1 Introduction

Non-surgical treatment of tumors has always been a major focus of clinical research. Traditional chemotherapy methods often cause negative effects on healthy tissues due to their lack of specificity, leading to severe adverse consequences and significantly diminishing the treatment efficacy (1). In recent years, the role of immune evasion mechanisms in the initiation and progression of tumors has been increasingly elucidated, and the development of immune checkpoint inhibitors—including Cytotoxic T-Lymphocyte Antigen 4 (CTLA-4) inhibitors, Programmed Cell Death Protein 1 (PD-1) inhibitors, and Programmed Cell Death Ligand 1 (PD-L1) inhibitors—has offered novel therapeutic options for patients (2). The efficacy of immunotherapy in various solid tumors has been validated through multiple clinical trials (3–7). Blocking the PD-1/PD-L1 axis enhances T lymphocyte response to tumor cells, thus accelerating immune-mediated tumor cell destruction (8). Tislelizumab, an anti-PD-1 monoclonal antibody, exhibits stronger affinity for PD-1 compared to nivolumab and pembrolizumab (5). Its dissociation rate is 50 times slower than nivolumab and 100 times slower than pembrolizumab, and it has demonstrated significant clinical efficacy in the treatment of various tumors (3–7). On March 14, 2024, Tislelizumab received FDA approval for the treatment of esophageal and gastric cancer. Although short-term clinical studies show that Tislelizumab has manageable safety (3–5), these studies are limited by small sample sizes, making it difficult to comprehensively assess its adverse effects. Since immune checkpoint inhibitors (ICIs) may lead to excessive immune activation, triggering immune-related adverse events (irAEs) and causing organ damage (9), it is necessary to assess the occurrence of irAEs during Tislelizumab treatment. The FDA Adverse Event Reporting System (FAERS) is a publicly accessible voluntary reporting system that contains a large number of drug-related adverse reaction records. As the largest pharmacovigilance database globally, FAERS is an important resource for identifying drug-related adverse reactions. This study aims to evaluate the safety profile of Tislelizumab and reveal adverse reactions not mentioned in the drug label. Additionally, we focus on comparing the differences in irAEs between Tislelizumab and other ICIs.

## 2 Materials and methods

### 2.1 Date source and de-duplications

**Figure 1:**
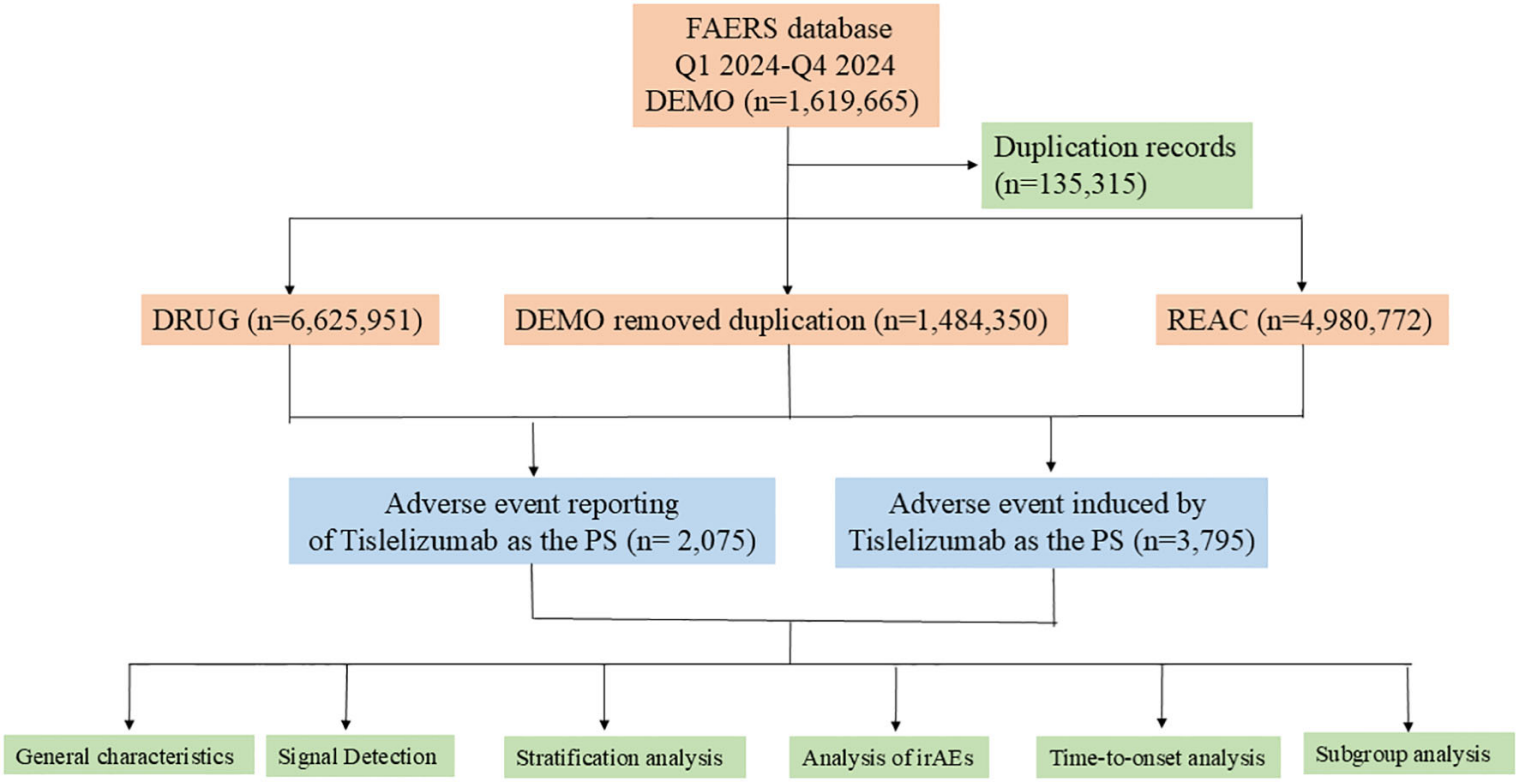
A flowchart illustrating the process of adverse event analysis for Tislelizumab using the FDA Adverse Event Reporting System database. PS, Primary Suspect Drug; irAEs, immune-related adverse events.

The data were sourced from the publicly accessible FAERS database, covering the period from the first quarter to the fourth quarter of 2024. Briefly, FAERS datafiles consist of seven datasets, including demographic and administrative information (DEMO), drug information (DRUG), adverse drug reaction information (REAC), patient outcome information (OUTC), information on report sources (RPSR), therapy start dates, and end dates for reported drugs (THER), as well as indications for drug administration (INDI). The data management process includes deduplication of duplicate reports and standardization of adverse reaction terminology. For reports with identical case identifiers (CASEIDs), the report with the latest FDA receipt date (FDA_DT) was retained. In cases where both CASEID and FDA_DT values matched, the report with the highest PRIMARYID (the unique identifier assigned to each report) was retained. Adverse reaction events were standardized using the MedDra dictionary (version 27.1), thereby enhancing the reliability of the statistical analysis. **Figure 1** provides a detailed process overview.

### 2.2 Statistical analysis

Descriptive analysis was used to present the characteristics of adverse reaction events associated with Tislelizumab. Disproportionality analysis was employed to assess the relationship between specific adverse reactions and Tislelizumab treatment. **Supplementary Table S1** provides detailed two-by-two contingency tables. Two disproportionality analysis methods were used to detect adverse reaction events related to Tislelizumab: the reporting odds ratio (ROR) and the Bayesian confidence propagation neural network (BCPNN). **Supplementary Table S2** describes the formulas and thresholds for both methods. To ensure the reliability of the results, adverse reaction events were considered positive if they were identified as such by both methods. The time interval between the occurrence of adverse reaction events (recorded in the DEMO file) and the start of Tislelizumab treatment (recorded in the THER file) was used to determine the latency period for the adverse reactions. The Weber distribution test was applied to examine the temporal variation in the incidence of adverse reactions. Cumulative incidences of AEs and irAEs were plotted using the Kaplan‐Meier method, and a log‐rank test was used to compare the cumulative incidences of AEs and irAEs in patients treated with Tislelizumab.

## 3 Results

### 3.1 General characteristics

From the first quarter to the fourth quarter of 2024, we included 2,075 cases of Tislelizumab, reporting 3,795 drug-related adverse reaction events (see **Table 1**). Among the included patients, 99.99% were from China. These cases were reported by 2,066 healthcare professionals (99.6%) and 9 non-healthcare professionals (0.4%). The primary reported indications were as follows: lung cancer (n = 605, 29.2%), esophageal cancer (n = 188, 9%), liver cancer (n = 148, 7.1%), nasopharyngeal cancer (n = 115, 5.5%), and gastric cancer (n = 69, 3.3%). Hospitalization was the most common serious adverse event (n = 647, 31.2%). Additionally, 18 deaths (0.8%) and 68 life-threatening cases (3.3%) were reported.

**Table 1:** Clinical characteristics of tislelizumab adverse event reports from the FAERS database (Q1 2024 – Q4 2024).

Characteristics	Case numbers	Case proportion (%)
Number of events	2,075	
Reported Countries		
China	2,073	99.99%
Korea, South	1	
Spain	1	
Reporter		
Healthcare professional	2,066	99.6%
Non-healthcare professional	9	0.4%
Reporting year		
2024Q1	83	4%
2024Q2	564	27.2%
2024Q3	701	33.8%
2024Q4	727	35%
Routes of administration		
Intravenous drip	1,897	91.4%
Intravenous bolus	2	0.1%
Unknown	176	8.5%
Indications		
Lung Neoplasms	605	29.2%
Esophageal Neoplasms	188	9%
Liver Neoplasms	148	7.1%
Nasopharyngeal Neoplasms	115	5.5%
Stomach Neoplasms	69	3.3%
Outcomes		
Death	18	0.8%
Disability	41	2%
Hospitalization (Initial or Prolonged)	647	31.2%
Life-Threatening	68	3.3%
Other Serious events	574	27.7%

### 3.2 Signal detection

**Figure 2:**
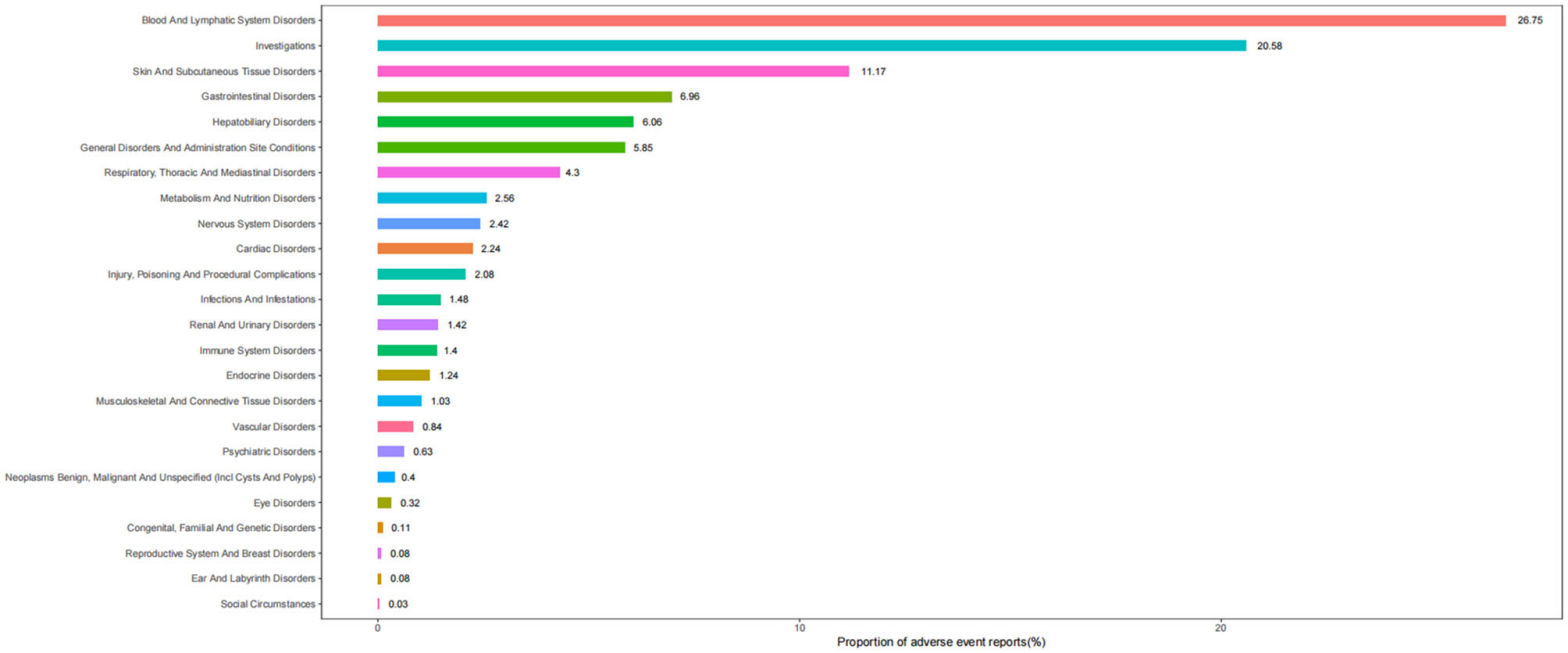
Proportion of adverse events categorized by system organ class for Tislelizumab.

**Figure 3:**
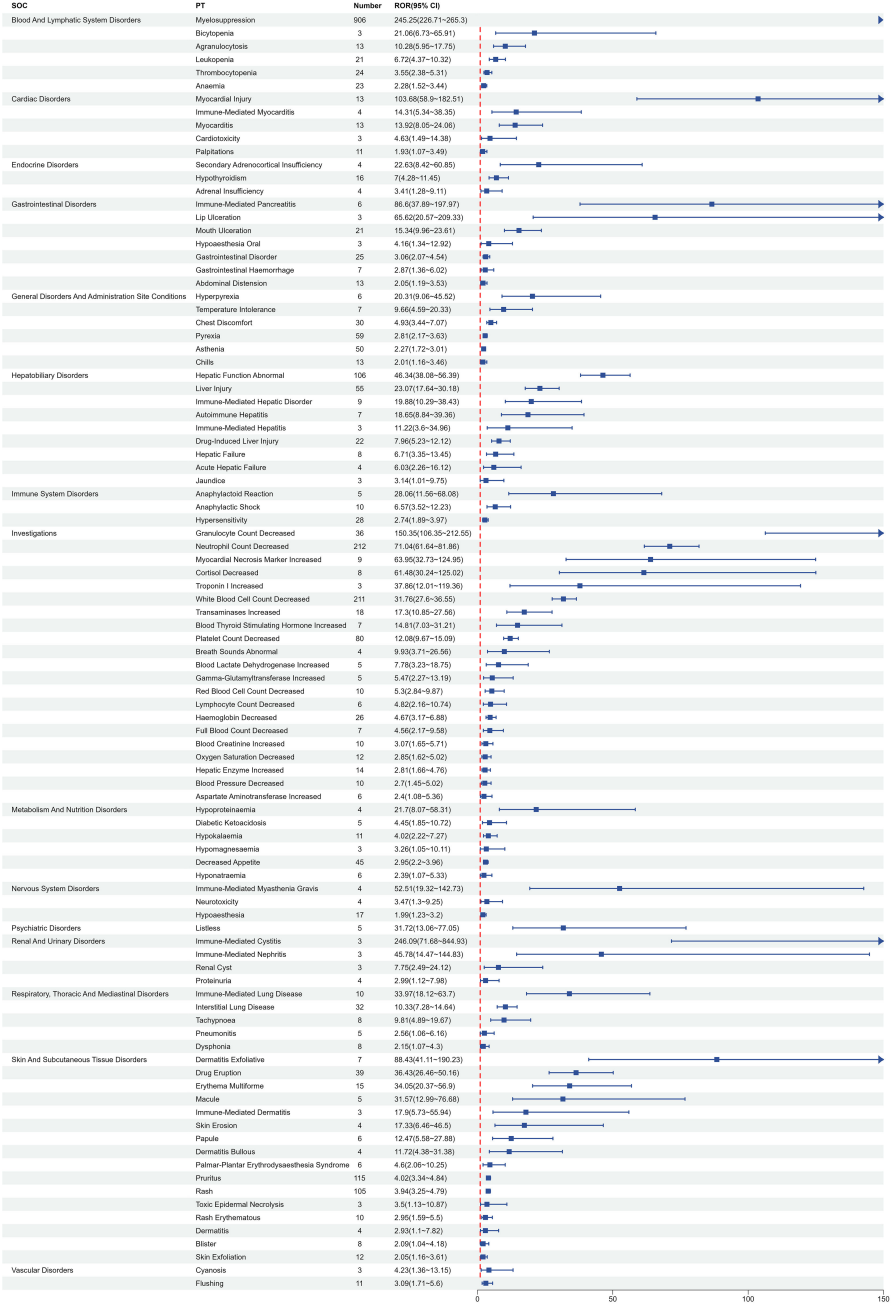
Signal strength of adverse events related to Tislelizumab at the PT level. SOC, System Organ Class; PT, Preferred term; Number, number of cases; ROR, reporting odds ratio; CI, confidence interval.

**Table 2** describes the signal strength of Tislelizumab at the SOC (System Organ Class) level. Adverse events associated with Tislelizumab were reported in 24 out of 27 SOCs. The SOCs that met the criteria of both algorithms include Blood and Lymphatic System Disorders, Investigations, Skin and Subcutaneous Tissue Disorders, Hepatobiliary Disorders, Metabolism and Nutrition Disorders, Cardiac Disorders, and Endocrine Disorders.The distribution of adverse events at the level of SOC is depicted in **Figure 2**. **Table 3** presents the preferred terms (PTs) with at least 3 cases and that meet the criteria of both algorithms, covering 97 PTs across 15 SOCs. The five most frequently reported PTs were as follows: Myelosuppression (n = 906), Neutrophil Count Decreased (n = 212), White Blood Cell Count Decreased (n = 211), Pruritus (n = 115), and Hepatic Function Abnormal(n = 106). The top five PTs based on significance, ranked by reporting odds ratio (ROR), were Immune-Mediated Cystitis (ROR = 246.09), Myelosuppression (ROR = 245.25), Granulocyte Count Decreased (ROR = 150.35), Myocardial Injury (ROR = 103.68), and Dermatitis Exfoliative (ROR = 88.43). Additionally, several potential adverse reactions not listed in the drug label were identified, including Palmar-Plantar Erythrodysaesthesia Syndrome, Immune-Mediated Cystitis, and Renal Cyst.All adverse events that met the criteria for a positive signal are shown in **Figure 3**.

**Table 2:** Signal strength of tislelizumab AEs across System Organ Classes (SOC) in the FAERS database.

System Organ Class (SOC)	Case numbers	ROR(95%CI)	IC(IC025)
Blood And Lymphatic System Disorders*	1015	20.37 (18.95 - 21.89)	3.91 (3.81)
Investigations*	781	4.12 (3.81 - 4.46)	1.8 (1.68)
Skin And Subcutaneous Tissue Disorders*	424	2.11 (1.9 - 2.33)	0.99 (0.84)
Gastrointestinal Disorders	264	0.81 (0.72 - 0.92)	-0.28 (-0.46)
Hepatobiliary Disorders*	230	7.04 (6.16 - 8.05)	2.73 (2.54)
General Disorders And Administration Site Conditions	222	0.3 (0.26 - 0.34)	-1.55 (-1.75)
Respiratory, Thoracic And Mediastinal Disorders	163	0.89 (0.76 - 1.04)	-0.16 (-0.39)
Metabolism And Nutrition Disorders*	97	1.29 (1.05 - 1.57)	0.35 (0.06)
Nervous System Disorders	92	0.32 (0.26 - 0.4)	-1.55 (-1.86)
Cardiac Disorders*	85	1.26 (1.01 - 1.56)	0.32 (0.01)
Injury, Poisoning And Procedural Complications	79	0.13 (0.1 - 0.16)	-2.77 (-3.1)
Infections And Infestations	56	0.22 (0.17 - 0.29)	-2.1 (-2.48)
Renal And Urinary Disorders	54	0.96 (0.73 - 1.25)	-0.06 (-0.45)
Immune System Disorders	53	1.13 (0.86 - 1.48)	0.17 (-0.22)
Endocrine Disorders*	47	4.06 (3.04 - 5.42)	2 (1.59)
Musculoskeletal And Connective Tissue Disorders	39	0.19 (0.14 - 0.26)	-2.34 (-2.8)
Vascular Disorders	32	0.46 (0.33 - 0.65)	-1.1 (-1.6)
Psychiatric Disorders	24	0.14 (0.1 - 0.22)	-2.74 (-3.31)
Neoplasms Benign, Malignant And Unspecified (Incl Cysts And Polyps)	15	0.23 (0.14 - 0.39)	-2.07 (-2.79)
Eye Disorders	12	0.15 (0.08 - 0.26)	-2.74 (-3.54)
Congenital, Familial And Genetic Disorders	4	0.4 (0.15 - 1.06)	-1.33 (-2.62)
Ear And Labyrinth Disorders	3	0.19 (0.06 - 0.59)	-2.39 (-3.83)
Reproductive System And Breast Disorders	3	0.14 (0.04 - 0.42)	-2.86 (-4.31)
Social Circumstances	1	0.05 (0.01 - 0.36)	-4.3 (-6.35)

Asterisks (*) indicate statistically significant signals in algorithm; ROR, reporting odds ratio; IC, information component; IC025, the lower limit of the 95% CI of the IC; CI, confidence interval; AEs, adverse events.

**Table 3:** Signal strength of reports of tislelizumab at the Preferred term (PT) level in FAERS database.

SOC	PT(Preferred Term)	a	ROR(95%Cl)	IC(IC025)
Blood And Lymphatic System Disorders	Myelosuppression	906	245.25 (226.71 - 265.3)	7.36 (7.24)
	Thrombocytopenia	24	3.55 (2.38 - 5.31)	1.82 (1.24)
	Anaemia	23	2.28 (1.52 - 3.44)	1.19 (0.59)
	Leukopenia	21	6.72 (4.37 - 10.32)	2.73 (2.12)
	Agranulocytosis	13	10.28 (5.95 - 17.75)	3.35 (2.57)
	Bicytopenia	3	21.06 (6.73 - 65.91)	4.37 (2.91)
Cardiac Disorders	Myocarditis	13	13.92 (8.05 - 24.06)	3.78 (3)
	Myocardial Injury	13	103.68 (58.9 - 182.51)	6.58 (5.78)
	Palpitations	11	1.93 (1.07 - 3.49)	0.95 (0.11)
	Immune-Mediated Myocarditis	4	14.31 (5.34 - 38.35)	3.82 (2.52)
	Cardiotoxicity	3	4.63 (1.49 - 14.38)	2.2 (0.76)
Endocrine Disorders	Hypothyroidism	16	7 (4.28 - 11.45)	2.8 (2.09)
	Adrenal Insufficiency	4	3.41 (1.28 - 9.11)	1.77 (0.47)
	Secondary Adrenocortical Insufficiency	4	22.63 (8.42 - 60.85)	4.48 (3.17)
Gastrointestinal Disorders	Gastrointestinal Disorder	25	3.06 (2.07 - 4.54)	1.61 (1.04)
	Mouth Ulceration	21	15.34 (9.96 - 23.61)	3.92 (3.3)
	Abdominal Distension	13	2.05 (1.19 - 3.53)	1.03 (0.26)
	Gastrointestinal Haemorrhage	7	2.87 (1.36 - 6.02)	1.52 (0.49)
	Immune-Mediated Pancreatitis	6	86.6 (37.89 - 197.97)	6.34 (5.21)
	Lip Ulceration	3	65.62 (20.57 - 209.33)	5.97 (4.48)
	Hypoaesthesia Oral	3	4.16 (1.34 - 12.92)	2.05 (0.6)
General Disorders And Administration Site Conditions	Pyrexia	59	2.81 (2.17 - 3.63)	1.47 (1.1)
	Asthenia	50	2.27 (1.72 - 3.01)	1.17 (0.77)
	Chest Discomfort	30	4.93 (3.44 - 7.07)	2.29 (1.77)
	Chills	13	2.01 (1.16 - 3.46)	1 (0.23)
	Temperature Intolerance	7	9.66 (4.59 - 20.33)	3.26 (2.23)
	Hyperpyrexia	6	20.31 (9.06 - 45.52)	4.32 (3.22)
Hepatobiliary Disorders	Hepatic Function Abnormal	106	46.34 (38.08 - 56.39)	5.45 (5.16)
	Liver Injury	55	23.07 (17.64 - 30.18)	4.48 (4.09)
	Drug-Induced Liver Injury	22	7.96 (5.23 - 12.12)	2.98 (2.37)
	Immune-Mediated Hepatic Disorder	9	19.88 (10.29 - 38.43)	4.29 (3.37)
	Hepatic Failure	8	6.71 (3.35 - 13.45)	2.74 (1.77)
	Autoimmune Hepatitis	7	18.65 (8.84 - 39.36)	4.2 (3.17)
	Acute Hepatic Failure	4	6.03 (2.26 - 16.12)	2.59 (1.29)
	Jaundice	3	3.14 (1.01 - 9.75)	1.65 (0.2)
	Immune-Mediated Hepatitis	3	11.22 (3.6 - 34.96)	3.48 (2.02)
Immune System Disorders	Hypersensitivity	28	2.74 (1.89 - 3.97)	1.44 (0.91)
	Anaphylactic Shock	10	6.57 (3.52 - 12.23)	2.71 (1.83)
	Anaphylactoid Reaction	5	28.06 (11.56 - 68.08)	4.78 (3.59)
Investigations	Neutrophil Count Decreased	212	71.04 (61.64 - 81.86)	6 (5.79)
	White Blood Cell Count Decreased	211	31.76 (27.6 - 36.55)	4.88 (4.67)
	Platelet Count Decreased	80	12.08 (9.67 - 15.09)	3.55 (3.23)
	Granulocyte Count Decreased	36	150.35 (106.35 - 212.55)	7.06 (6.56)
	Haemoglobin Decreased	26	4.67 (3.17 - 6.88)	2.21 (1.65)
	Transaminases Increased	18	17.3 (10.85 - 27.56)	4.09 (3.42)
	Hepatic Enzyme Increased	14	2.81 (1.66 - 4.76)	1.49 (0.74)
	Oxygen Saturation Decreased	12	2.85 (1.62 - 5.02)	1.51 (0.7)
	Blood Pressure Decreased	10	2.7 (1.45 - 5.02)	1.43 (0.55)
	Red Blood Cell Count Decreased	10	5.3 (2.84 - 9.87)	2.4 (1.52)
	Blood Creatinine Increased	10	3.07 (1.65 - 5.71)	1.61 (0.74)
	Myocardial Necrosis Marker Increased	9	63.95 (32.73 - 124.95)	5.93 (4.99)
	Cortisol Decreased	8	61.48 (30.24 - 125.02)	5.87 (4.89)
	Blood Thyroid Stimulating Hormone Increased	7	14.81 (7.03 - 31.21)	3.87 (2.84)
	Full Blood Count Decreased	7	4.56 (2.17 - 9.58)	2.18 (1.16)
	Aspartate Aminotransferase Increased	6	2.4 (1.08 - 5.36)	1.26 (0.17)
	Lymphocyte Count Decreased	6	4.82 (2.16 - 10.74)	2.26 (1.17)
	Gamma-Glutamyltransferase Increased	5	5.47 (2.27 - 13.19)	2.45 (1.26)
	Blood Lactate Dehydrogenase Increased	5	7.78 (3.23 - 18.75)	2.95 (1.77)
	Breath Sounds Abnormal	4	9.93 (3.71 - 26.56)	3.3 (2)
	Troponin I Increased	3	37.86 (12.01 - 119.36)	5.2 (3.73)
Metabolism And Nutrition Disorders	Decreased Appetite	45	2.95 (2.2 - 3.96)	1.55 (1.12)
	Hypokalaemia	11	4.02 (2.22 - 7.27)	2 (1.16)
	Hyponatraemia	6	2.39 (1.07 - 5.33)	1.25 (0.16)
	Diabetic Ketoacidosis	5	4.45 (1.85 - 10.72)	2.15 (0.97)
	Hypoproteinaemia	4	21.7 (8.07 - 58.31)	4.42 (3.11)
	Hypomagnesaemia	3	3.26 (1.05 - 10.11)	1.7 (0.25)
Nervous System Disorders	Hypoaesthesia	17	1.99 (1.23 - 3.2)	0.99 (0.3)
	Immune-Mediated Myasthenia Gravis	4	52.51 (19.32 - 142.73)	5.66 (4.34)
	Neurotoxicity	4	3.47 (1.3 - 9.25)	1.79 (0.5)
Psychiatric Disorders	Listless	5	31.72 (13.06 - 77.05)	4.95 (3.76)
Renal And Urinary Disorders	Proteinuria	4	2.99 (1.12 - 7.98)	1.58 (0.28)
	Renal Cyst	3	7.75 (2.49 - 24.12)	2.95 (1.5)
	Immune-Mediated Cystitis	3	246.09 (71.68 - 844.93)	7.7 (6.11)
	Immune-Mediated Nephritis	3	45.78 (14.47 - 144.83)	5.47 (3.99)
Respiratory, Thoracic And Mediastinal Disorders	Interstitial Lung Disease	32	10.33 (7.28 - 14.64)	3.35 (2.84)
	Immune-Mediated Lung Disease	10	33.97 (18.12 - 63.7)	5.05 (4.16)
	Dysphonia	8	2.15 (1.07 - 4.3)	1.1 (0.14)
	Tachypnoea	8	9.81 (4.89 - 19.67)	3.28 (2.31)
	Pneumonitis	5	2.56 (1.06 - 6.16)	1.35 (0.17)
Skin And Subcutaneous Tissue Disorders	Pruritus	115	4.02 (3.34 - 4.84)	1.97 (1.7)
	Rash	105	3.94 (3.25 - 4.79)	1.95 (1.66)
	Drug Eruption	39	36.43 (26.46 - 50.16)	5.13 (4.67)
	Erythema Multiforme	15	34.05 (20.37 - 56.9)	5.05 (4.32)
	Skin Exfoliation	12	2.05 (1.16 - 3.61)	1.03 (0.23)
	Rash Erythematous	10	2.95 (1.59 - 5.5)	1.56 (0.69)
	Blister	8	2.09 (1.04 - 4.18)	1.06 (0.1)
	Dermatitis Exfoliative	7	88.43 (41.11 - 190.23)	6.37 (5.31)
	Palmar-Plantar Erythrodysaesthesia Syndrome	6	4.6 (2.06 - 10.25)	2.19 (1.1)
	Papule	6	12.47 (5.58 - 27.88)	3.63 (2.53)
	Macule	5	31.57 (12.99 - 76.68)	4.95 (3.75)
	Dermatitis Bullous	4	11.72 (4.38 - 31.38)	3.54 (2.24)
	Skin Erosion	4	17.33 (6.46 - 46.5)	4.1 (2.79)
	Dermatitis	4	2.93 (1.1 - 7.82)	1.55 (0.25)
	Immune-Mediated Dermatitis	3	17.9 (5.73 - 55.94)	4.14 (2.69)
	Toxic Epidermal Necrolysis	3	3.5 (1.13 - 10.87)	1.8 (0.36)
Vascular Disorders	Flushing	11	3.09 (1.71 - 5.6)	1.62 (0.79)
	Cyanosis	3	4.23 (1.36 - 13.15)	2.08 (0.63)

ROR, reporting odds ratio; IC025, the lower limit of the 95% CI of the IC; CI, confidence interval; PT, preferred term.

### 3.3 Subgroup analysis

We conducted a subgroup analysis of several common indications for Tislelizumab treatment in the FAERS database. The results indicated that, under the criteria of both algorithms, the most common adverse reactions in the lung cancer group were myelosuppression (n = 260), white blood cell count decreased (n = 65), neutrophil count decreased (n = 60), rash (n = 35), and pruritus (n = 32). In the esophageal cancer group, the most common adverse reactions were myelosuppression (n = 94), neutrophil count decreased (n = 21), white blood cell count decreased (n = 20), platelet count decreased (n = 11), and rash (n = 7). For the liver cancer group, the most common adverse reactions were myelosuppression (n = 65), hepatic function abnormal (n = 11), rash (n = 7), pruritus (n = 7), drug eruption (n = 7), and neutrophil count decreased (n = 6). In the nasopharyngeal cancer group, the most common adverse reactions were myelosuppression (n = 54), neutrophil count decreased (n = 24), rash (n = 9), and pruritus (n = 9). The most common adverse reactions in the gastric cancer group were myelosuppression (n = 35), neutrophil count decreased (n = 8), white blood cell count decreased (n = 7), pruritus (n = 5), and rash (n = 4). Myelosuppression was the most frequently reported adverse event across all subgroups, while neutrophil count decreased, rash, and pruritus occurred in all subgroups.Specific details are provided in **Supplementary Tables S3-S7**.

### 3.4 Analysis of irAEs

**Figure 4:**
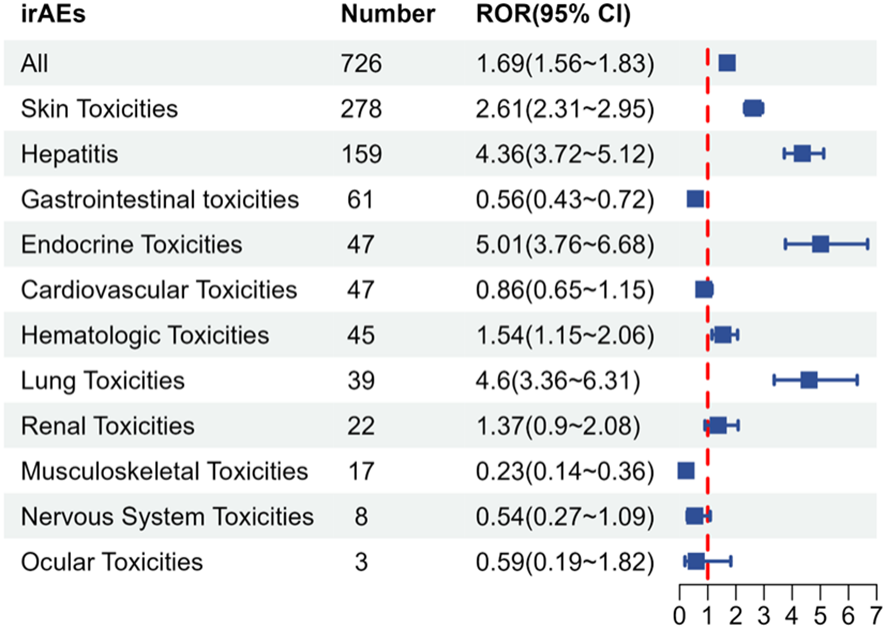
irAE signals associated with Tislelizumab. irAE, immune-related adverse event; Number, number of cases; ROR, reporting odds ratio; CI, confidence interval.

The compilation of immune-related adverse events (irAEs) was obtained from the study conducted by Chen Chen et al. (10) and categorized into 11 groups, including Skin Toxicities, Gastrointestinal Toxicities, Hepatitis, Endocrine Toxicities, Lung Toxicities, Musculoskeletal Toxicities, Cardiovascular Toxicities, Hematologic Toxicities, Renal Toxicities, Nervous System Toxicities, and Ocular Toxicities. Overall, Tislelizumab demonstrated a significant signal for irAEs (n = 726, ROR = 1.69), with a lower ROR compared to other immune checkpoint inhibitors (**Supplementary Table S8**). When irAEs were analyzed across the 11 categories, five positive signals were identified (**Figure 4**), namely: Skin Toxicities (n = 278, ROR = 2.61), Hepatitis (n = 159, ROR = 4.36), Endocrine Toxicities (n = 47, ROR = 5.01), Hematologic Toxicities (n = 45, ROR = 1.54), and Lung Toxicities (n = 39, ROR = 4.6).Specific details are presented in **Supplementary Table S9**.

### 3.5 Time to onset analysis for AEs and irAEs associated with tislelizumab

**Figure 5:**
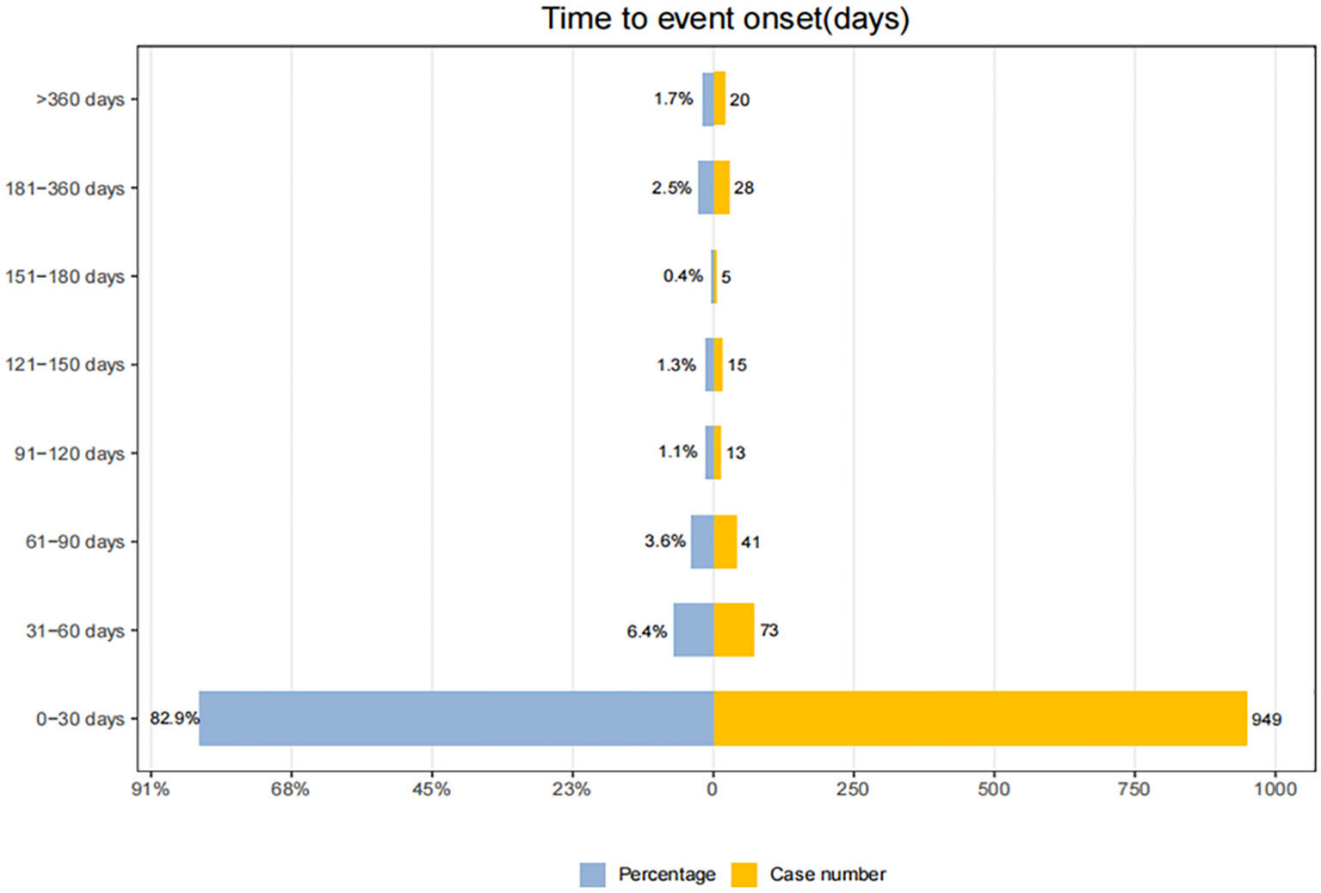
Time to onset of adverse events associated with Tislelizumab.

**Figure 6:**
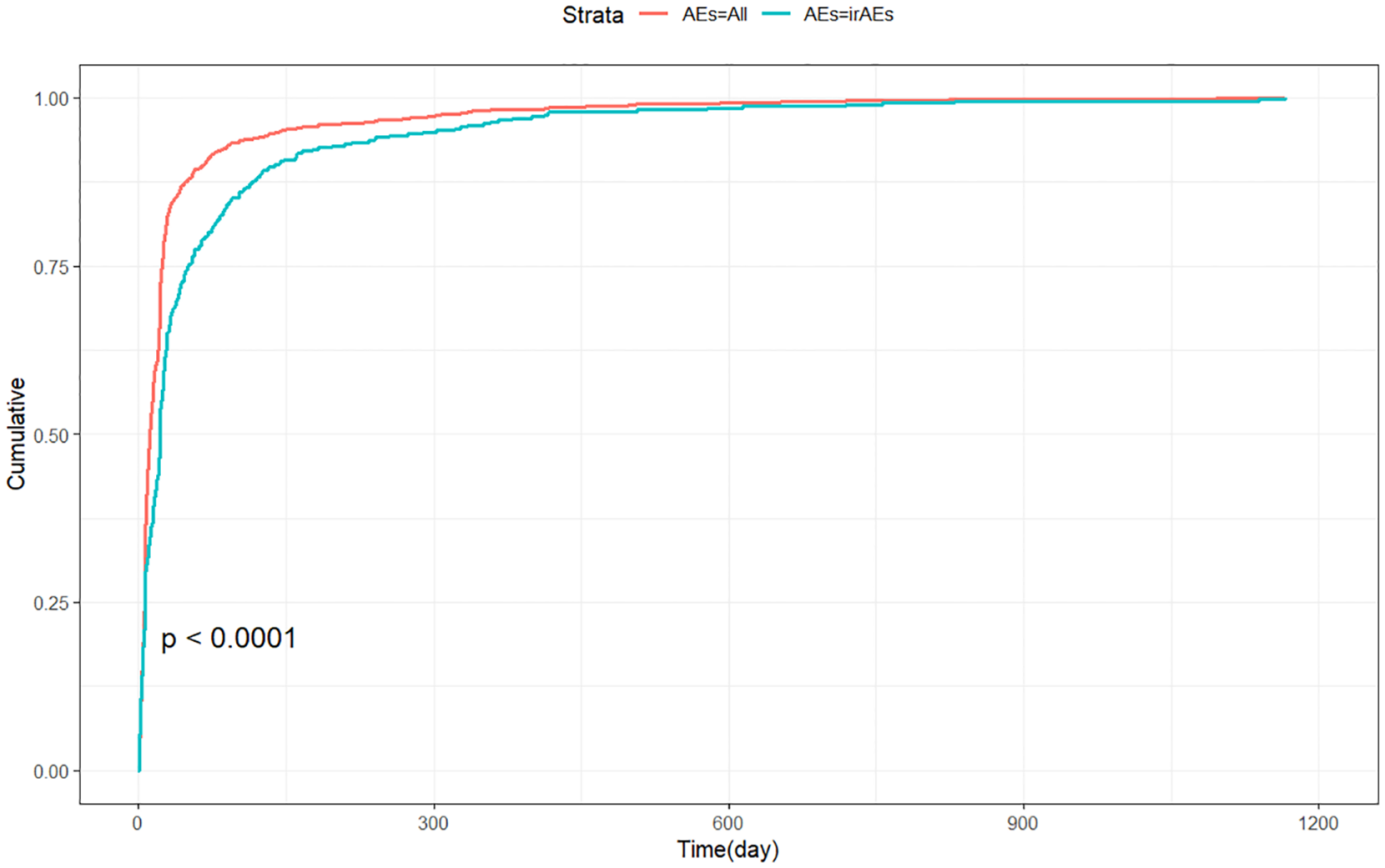
Cumulative incidence of adverse events associated with Tislelizumab over time. AEs, adverse events; irAEs, immune-related adverse events.

Adverse events (AEs) associated with Tislelizumab most commonly occurred within the first month of treatment.The temporal distribution of these events is specifically depicted in **Figure 5**. The median time-to-onset of Tislelizumab was 11 days (IQR: 6–24 days), while for immune-related adverse events (irAEs) associated with Tislelizumab, the median time-to-onset was 21 days (IQR: 6–50.5 days). The cumulative incidence curves for all adverse events (AEs) and immune-related adverse events (irAEs) associated with Tislelizumab are shown in the **Figure 6**. Additionally, Weber distribution analysis demonstrated that both exhibited an early failure mode. Detailed parameters of the Weber analysis are listed in the **Table 4**.

**Table 4:** Weibull distribution analysis for AEs and irAEs associated with tislelizumab.

AEs	TTO (days)		Weibull distribution			P
	Case reports	Median (d) (IQR)	Scale parameter: α (95%CI)	Shape parameter: β (95%CI)	Type	
All	1144	11 (6-24)	24.41 (22.19-26.62)	0.679 (0.653-0.706)	Early failure	P<0.001
irAEs	391	21 (6-50.5)	42.11 (35.34-48.87)	0.655 (0.608-0.701)	Early failure	

TTO,time to onset; CI, confidence interval; IQR, interquartile range.

### 3.6 Sensitivity analysis

Tislelizumab is frequently used in combination with several other drugs, such as Cisplatin, Oxaliplatin, Docetaxel, Gemcitabine, and Pemetrexed. After excluding reports involving combinations with these five drugs, we included 1,424 reports, which encompassed 2,690 adverse event reports. The adverse reactions that still met the positive criteria included Palmar-Plantar Erythrodysesthesia Syndrome, Immune-Mediated Cystitis, and Renal Cyst (**Supplementary Table S10**).

## 4 Discussion

Previous clinical studies have demonstrated that Tislelizumab shows favorable progression-free survival (PFS) and objective response rates (ORR) in the treatment of non-small cell lung cancer (5). In China, Tislelizumab has been approved for the treatment of advanced squamous and non-squamous (NSCLCs), hepatocellular carcinoma (HCC), esophageal squamous cell carcinoma (ESCC), urothelial carcinoma(UC), classical Hodgkin’s lymphoma (cHL) (11, 12). Among the medication records included in this study, the most common indications were not the FDA-approved esophageal cancer and gastric cancer, with lung cancer being the most frequent indication, accounting for about one-third of the reported cases. This is primarily due to the fact that most of the reports included in this study originated from China. Tislelizumab combined with chemotherapy has shown promising clinical results in the treatment of various cancer types. For instance, tislelizumab combined with gemcitabine plus cisplatin has demonstrated excellent clinical efficacy in the treatment of urothelial carcinoma(UC) (4). To minimize outcome bias caused by common clinical combination therapies, we excluded records involving combinations with Cisplatin, Oxaliplatin, Docetaxel, Gemcitabine, Pemetrexed. Through the analysis of the FAERS database, we identified several adverse reactions that are already listed in the Tislelizumab drug label, such as myelosuppression, hepatic function abnormalities, pruritus, rash, and exfoliative dermatitis. Additionally, we discovered some novel adverse reactions that are not listed in the drug label, including palmar-plantar erythrodysaesthesia syndrome, immune-mediated cystitis, and renal cysts.

In previous studies, Chen et al. identified various hematological adverse events associated with the use of immune checkpoint inhibitors (ICIs) through the analysis of the FAERS database (10). A systematic review of clinical trials suggested that the most common adverse reactions in the Tislelizumab treatment group were hematological parameter reductions (anemia, neutropenia, thrombocytopenia, and leukopenia), which is consistent with our findings (5). The exact mechanism of hematological adverse events related to Tislelizumab remains unclear; Excessive T cell activation and the potential removal of immune checkpoints may partially explain the mechanism underlying the occurrence of these adverse events (13). Comprehensive hematological monitoring should be performed for patients, including bone marrow biopsy if necessary. In terms of treatment, 1-2 mg/kg of prednisone daily may be used as needed, and blood transfusion support should be provided if necessary (13). The standard treatment regimen for Tislelizumab-related hematological disorders still requires further investigation.

Another notable adverse reaction is hepatic dysfunction. A phase III clinical study comparing the efficacy and safety of Tislelizumab and sorafenib in the treatment of hepatocellular carcinoma reported that elevated ALT, AST, and serum bilirubin levels were the most common adverse reactions in the Tislelizumab group, which is consistent with our analysis (7). The non-specific activation of the immune system related to Tislelizumab not only enhances immune-mediated tumor cell destruction but may also cause immune-related damage to hepatocytes and other normal tissues. The hemi-antigen hypothesis suggests that reactive metabolites bind to cellular proteins to form novel antigens called “haptens.” These “haptens” are presented to major histocompatibility complex molecules on antigen-presenting cells, activating cytotoxic T lymphocytes, B cells, and natural killer cells, which can lead to immune damage to hepatocytes (14, 15). Regular monitoring of liver function in patients receiving Tislelizumab treatment should be conducted, and immune-mediated hepatotoxicity should be managed with dose reduction, discontinuation, or immunosuppressive therapy when necessary (16).

Consistent with the drug label, our analysis identified dermatological adverse reactions such as pruritus, rash, and exfoliative dermatitis. In a clinical study by Qin et al., 35 out of 338 patients treated with Tislelizumab experienced pruritus (10.4%), and 34 experienced a rash (10.1%) (7). Dermatological adverse reactions can cause significant physical and psychological distress, leading to an increased risk of treatment discontinuation. Early diagnosis and timely management of these skin reactions may help patients continue receiving Tislelizumab treatment, resulting in better clinical outcomes. For example, topical corticosteroids can be used to alleviate pruritus (17). Notably, we identified a dermatological adverse reaction not listed on the drug label—Palmar-Plantar Erythrodysaesthesia Syndrome (PPES). Consistent with our findings, Qin et al. reported one case of PPES in the Tislelizumab treatment group (7). The mechanism underlying Tislelizumab-related PPES remains unclear, but the effectiveness of dexamethasone in treating PPES suggests that excessive immune system activation may be part of its pathogenesis (18). Although it is rarely life-threatening, it significantly affects the patient’s quality of life. Dose adjustment is effective in treating PPES. Additionally, some preventive strategies, such as the use of urea-based creams twice daily, have been shown in a randomized controlled study to effectively prevent PPES (18). Further research is needed to understand the pathogenesis and management of Tislelizumab-related PPES.

Our analysis found that the highest signal value for an adverse reaction was immune-mediated cystitis, which is not listed on the Tislelizumab drug label. Previous case studies have reported that Tislelizumab may induce immune-related ureteritis/cystitis (19, 20). High levels of PD-L1 expression were found in the bladder tissue of patients with severe cystitis (21). Therefore, Tislelizumab-induced cytotoxic T cell activation may not only target cancer cells but also attack normal urothelial cells expressing PD-L1. Clinically, immune-mediated ureteritis/cystitis may present as paroxysmal lower abdominal pain or bladder irritation symptoms. Urine analysis often shows elevated red blood cell and white blood cell counts, proteinuria, and negative urine cultures repeatly. Cystoscopy may reveal revealed diffused redness of the bladder mucosa, while CT scans can show ureteral dilation and bladder wall thickening. During Tislelizumab treatment, any new urinary symptoms or abnormal urine analysis should raise a high suspicion for immune-mediated ureteritis/cystitis. Regular urine analysis, renal function tests, and urinary imaging are helpful for early identification of immune-mediated ureteritis/cystitis, allowing effective relief of urinary symptoms and immune-related urinary tract damage through glucocorticoid treatment while avoiding unnecessary antibiotic use (19, 20). Furthermore, we identified a potential adverse reaction of Tislelizumab—renal cysts—which had not been previously reported. The potential mechanism by which Tislelizumab causes renal cysts remains unclear. Previous studies suggest that kidney injury can accelerate the growth of renal cysts (22), and we hypothesize that renal cysts may fall under the category of immune-mediated renal Toxicities, which requires further investigation to reveal its mechanism.

We further investigated the profile of immune-related adverse events associated with Tislelizumab. Compared to Anti-PD-1 (Nivolumab: OR 2.21; Pembrolizumab: OR 2.35; Cemiplimab: OR 2.42), Anti-PD-L1 (Atezolizumab: OR 2.27; Durvalumab: OR 3.84), Anti-CTLA-4 (Ipilimumab: OR 3.01; Tremelimumab: OR 4.52), and Combination therapy (Ipilimumab + Nivolumab: OR 4.80; Ipilimumab + Pembrolizumab: OR 7.77; Durvalumab + Tremelimumab: OR 7.03) (10), Tislelizumab exhibited a lower signal for irAEs (ROR 1.69). Our findings suggest that Tislelizumab offers better safety compared to other immune checkpoint inhibitors (ICIs) (23). For patient populations at high risk of immune-related adverse events, an immune therapy regimen based on Tislelizumab may be superior to other ICIs and could become the preferred option. Combining immune checkpoint inhibitors (ICIs) may help overcome resistance pathways and improve sensitivity to PD-1/PD-L1 therapy (24, 25). Furthermore, this combination could enhance efficacy while minimizing drug toxicity by reducing dosages and shortening the treatment duration. In a previous phase 1/2 clinical study, BGB-A333 (a novel PD-L1 inhibitor) combined with Tislelizumab demonstrated promising antitumor effects without increasing the risk of irAEs (26). Further research is urgently needed to explore immune checkpoint inhibitor (ICI) combination regimens based on Tislelizumab. We also conducted a temporal analysis of adverse events, which highlighted the importance of early detection and management of related adverse events by clinicians during the first month of Tislelizumab treatment.

This study has several limitations. Firstly, the FAERS is a spontaneous reporting system that compiles data from multiple countries and various professionals, which may sometimes be incomplete or inaccurate. Secondly, certain confounding factors, such as drug dosage, duration of use, and combination therapies, may introduce bias into the analysis. Fortunately, we excluded cases involving the five most common combination drugs with Tislelizumab. Moreover, the majority of the study data was sourced from China, which may lead to reporting bias. Future research should incorporate data from multiple countries to enhance the generalizability of the findings. Lastly, disproportionality analysis can only assess signal strength, and thus, this study could not establish a causal relationship between the target drug and adverse events. Further experimental research is needed to validate these risks.

## 5 Conclusion

In this study, we conducted a comprehensive analysis of Tislelizumab-related adverse events using the FAERS database. We identified adverse events that are already listed on the drug label, and notably, we also uncovered potential adverse events not mentioned on the label, such as palmar-plantar erythrodysaesthesia syndrome, renal cysts, immune-mediated cystitis. This study overcomes the limitation of insufficient sample size in clinical trials, providing clinicians with essential safety information when prescribing Tislelizumab. It also emphasizes the need for close monitoring of potential adverse reactions in patients. Further validation is required through the collection of hospital data or the use of other adverse drug reaction databases, and prospective clinical studies are needed to clarify the causal relationship between Tislelizumab and these adverse events. Furthermore, we highlighted the lower immune-related adverse event signal associated with Tislelizumab compared to other ICIs. This large-scale real-world study systematically evaluated the overall safety of Tislelizumab, highlighting the urgent need to develop clinical predictive models for adverse reactions to further enhance medication safety and promote personalized treatment. The relatively low risk of irAEs associated with Tislelizumab makes it a potential candidate for combination therapy with other ICIs. Therefore, novel ICI combination strategies based on Tislelizumab are expected to provide new therapeutic hope for patients burdened by irAEs. Further clinical trials are warranted to systematically evaluate the efficacy and safety of such combination therapies.

## Data Availability

The original contributions presented in the study are included in the article/**Supplementary Material**, further inquiries can be directed to the corresponding authors.

## References

[1] Sharma P Mehta M Dhanjal DS Kaur S Gupta G Singh H. Emerging trends in the novel drug delivery approaches for the treatment of lung cancer. Chem Biol Interact. (2019) 309:108720. doi: 10.1016/j.cbi.2019.06.033 31226287

[2] Liu S-Y Wu Y-L. Tislelizumab: an investigational anti-PD-1 antibody for the treatment of advanced non-small cell lung cancer (NSCLC). Expert Opin Investig Drugs. (2020) 29:1355–64. doi: 10.1080/13543784.2020.1833857 33044117

[3] Jiang Q Liu W Zeng X Zhang C Du Y Zeng L. Safety and efficacy of tislelizumab plus chemotherapy versus chemotherapy alone as neoadjuvant treatment for patients with locally advanced gastric cancer: real-world experience with a consecutive patient cohort. Front Immunol. (2023) 14:1122121. doi: 10.3389/fimmu.2023.1122121 37215127 PMC10195027

[4] Zhang J Yang M Wei D Zhang D Chen Z Zhu H. The efficacy and safety of tislelizumab combined with gemcitabine plus cisplatin in the treatment of postoperative patients with muscle-invasive upper tract urothelial carcinoma. BMC Cancer. (2024) 24:202. doi: 10.1186/s12885-024-11919-1 38350941 PMC10863243

[5] Daei Sorkhabi A ZareDini M Fazlollahi A Sarkesh A Naseri A Mousavi SE. The safety and efficacy of tislelizumab, alone or in combination with chemotherapy, for the treatment of non-small cell lung cancer: a systematic review of clinical trials. BMC Pulm Med. (2023) 23:495. doi: 10.1186/s12890-023-02755-3 38066549 PMC10704633

[6] Zhou C Huang D Fan Y Yu X Liu Y Shu Y. Tislelizumab versus docetaxel in patients with previously treated advanced NSCLC (RATIONALE-303): A phase 3, open-label, randomized controlled trial. J Thorac Oncol Off Publ Int Assoc Study Lung Cancer. (2023) 18:93–105. doi: 10.1016/j.jtho.2022.09.217 36184068

[7] Qin S Kudo M Meyer T Bai Y Guo Y Meng Z. Tislelizumab vs sorafenib as first-line treatment for unresectable hepatocellular carcinoma: A phase 3 randomized clinical trial. JAMA Oncol. (2023) 9. doi: 10.1001/jamaoncol.2023.4003 PMC10557031 37796513

[8] Anand K Sahu G Burns E Ensor A Ensor J Pingali SR. Mycobacterial infections due to PD-1 and PD-L1 checkpoint inhibitors. ESMO Open. (2020) 5:e000866. doi: 10.1136/esmoopen-2020-000866 32817069 PMC7437685

[9] Yang F Shay C Abousaud M Tang C Li Y Qin Z. Patterns of toxicity burden for FDA-approved immune checkpoint inhibitors in the United States. J Exp Clin Cancer Res CR. (2023) 42:4. doi: 10.1186/s13046-022-02568-y 36600271 PMC9814433

[10] Chen C Wu B Zhang C Xu T. Immune-related adverse events associated with immune checkpoint inhibitors: An updated comprehensive disproportionality analysis of the FDA adverse event reporting system. Int Immunopharmacol. (2021) 95:107498. doi: 10.1016/j.intimp.2021.107498 33725634

[11] Shen L Guo J Zhang Q Pan H Yuan Y Bai Y. Tislelizumab in Chinese patients with advanced solid tumors: an open-label, non-comparative, phase 1/2 study. J Immunother Cancer. (2020) 8:e000437. doi: 10.1136/jitc-2019-000437 32561638 PMC7304812

[12] Lee A Keam SJ. Tislelizumab: first approval. Drugs. (2020) 80:617–24. doi: 10.1007/s40265-020-01286-z 32185681

[13] Kroll MH Rojas-Hernandez C Yee C. Hematologic complications of immune checkpoint inhibitors. Blood. (2022) 139:3594–604. doi: 10.1182/blood.2020009016 PMC9227102 34610113

[14] David S Hamilton JP. Drug-induced liver injury. US Gastroenterol Hepatol Rev. (2010) 6:73–80. doi: 10.3389/fimmu.2022.1085057 21874146 PMC3160634

[15] Uetrecht J. Idiosyncratic drug reactions: past, present, and future. Chem Res Toxicol. (2008) 21:84–92. doi: 10.1021/tx700186p 18052104

[16] Peeraphatdit TB Wang J Odenwald MA Hu S Hart J Charlton MR. Hepatotoxicity from immune checkpoint inhibitors: A systematic review and management recommendation. Hepatol Baltim Md. (2020) 72:315–29. doi: 10.1002/hep.31227 32167613

[17] Quach HT Johnson DB LeBoeuf NR Zwerner JP Dewan AK. Cutaneous adverse events caused by immune checkpoint inhibitors. J Am Acad Dermatol. (2021) 85:956–66. doi: 10.1016/j.jaad.2020.09.054 34332798

[18] Miller KK Gorcey L McLellan BN. Chemotherapy-induced hand-foot syndrome and nail changes: a review of clinical presentation, etiology, pathogenesis, and management. J Am Acad Dermatol. (2014) 71:787–94. doi: 10.1016/j.jaad.2014.03.019 24795111

[19] Zhou Q Qin Z Yan P Wang Q Qu J Chen Y. Immune-related adverse events with severe pain and ureteral expansion as the main manifestations: a case report of tislelizumab-induced ureteritis/cystitis and review of the literature. Front Immunol. (2023) 14:1226993. doi: 10.3389/fimmu.2023.1226993 37869004 PMC10587548

[20] Ji J Lai C-H Zhang X Hu H. Immune-related adverse events with renal colic as the main manifestation: a case report of sintilimab-induced ureteritis/cystitis treated by ureteral stent and review of the literature. Front Immunol. (2024) 15:1501415. doi: 10.3389/fimmu.2024.1501415 39763683 PMC11700999

[21] Li J Yu Y-F Qi X-W Du Y Li C-Q. Immune-related ureteritis and cystitis induced by immune checkpoint inhibitors: Case report and literature review. Front Immunol. (2022) 13:1051577. doi: 10.3389/fimmu.2022.1051577 36685488 PMC9853439

[22] Kleczko EK Nguyen DT Marsh KH Bauer CD Li AS Monaghan M-LT. Immune checkpoint activity regulates polycystic kidney disease progression. JCI Insight. (2023) 8:e161318. doi: 10.1172/jci.insight.161318 37345660 PMC10371237

[23] Chennamadhavuni A Abushahin L Jin N Presley CJ Manne A. Risk factors and biomarkers for immune-related adverse events: A practical guide to identifying high-risk patients and rechallenging immune checkpoint inhibitors. Front Immunol. (2022) 13:779691. doi: 10.3389/fimmu.2022.779691 35558065 PMC9086893

[24] Wu M Huang Q Xie Y Wu X Ma H Zhang Y. Improvement of the anticancer efficacy of PD-1/PD-L1 blockade via combination therapy and PD-L1 regulation. J Hematol OncolJ Hematol Oncol. (2022) 15:24. doi: 10.1186/s13045-022-01242-2 35279217 PMC8917703

[25] Li Z Sun G Sun G Cheng Y Wu L Wang Q. Various uses of PD1/PD-L1 inhibitor in oncology: opportunities and challenges. Front Oncol. (2021) 11:771335. doi: 10.3389/fonc.2021.771335 34869005 PMC8635629

[26] Desai J Fong P Moreno V Frentzas S Meniawy T Markman B. A Phase 1/2 study of the PD-L1 inhibitor, BGB-A333, alone and in combination with the PD-1 inhibitor, tislelizumab, in patients with advanced solid tumours. Br J Cancer. (2023) 128:1418–28. doi: 10.1038/s41416-022-02128-3 PMC10070264 36797356

