# Supplementary material for: Post-marketing safety concerns with Tislelizumab: a disproportionality analysis of the FDA adverse event reporting system
Source: Front Immunol. 2025 May 26;16:1596842. doi: 10.3389/fimmu.2025.1596842 (PMC12146294; doi:10.3389/fimmu.2025.1596842)
Supplement: Supplementary file 1 [file DataSheet1.pdf]

Supplementary Material

Supplementary Table 1

Two-by-two contingency table for disproportionality analyses.

	Target AEs	Other AEs	Total
Target drug	a	b	a+b
Other drugs	c	d	c+d
Total	a+c	b+d	a+b+c+d

Abbreviation: AEs, adverse events; a, number of reports containing both the target drug and target adverse drug reaction; b, number of reports containing other adverse drug reaction of the target drug; c, number of reports containing the target adverse drug reaction of other drugs; d, number of reports containing other drugs and other adverse drug reactions.

Supplementary Table 2

Two major algorithms used for signal detection.

Algorithms	Equation	Criteria
ROR	ROR=ad/b/c<br>95%CI=$e^{\ln(ROR)\pm 1.96(1/a+1/b+1/c+1/d)^{\wedge}0.5}$	lower limit of 95% CI>1, N≥3
BCPNN	IC=$\log_2$a(a+b+c+d)(a+c)(a+b)<br>95%CI= E(IC) ± 2V(IC)^0.5	IC025>0

Abbreviation: a, number of reports containing both the target drug and target adverse drug reaction; b, number of reports containing other adverse drug reaction of the target drug; c, number of reports containing the target adverse drug reaction of other drugs; d, number of reports containing other drugs and other adverse drug reactions. 95%CI, 95% confidence interval; N, the number of reports; IC, information component; IC025, the lower limit of 95% CI of the IC; E(IC), the IC expectations; V(IC), the variance of IC;

Supplementary Table 3 Signal strength of tislelizumab in patients in the Lung Neoplasms group from FAERS data

SOC	PT(Preferred Term)	a	ROR(95%Cl)	IC(IC025)
Blood And Lymphatic System Disorders	Myelosuppression	260	57.99 (48.14 - 69.85)	4.49 (4.25)
Cardiac Disorders	Myocarditis	5	2.98 (1.2 - 7.39)	1.51 (0.28)
	Myocardial Injury	3	10.87 (3.06 - 38.59)	3.15 (1.53)
Gastrointestinal Disorders	Mouth Ulceration	8	6.99 (3.3 - 14.77)	2.61 (1.58)
	Gastrointestinal Disorder	7	4.92 (2.25 - 10.78)	2.17 (1.09)

	Abdominal Distension	7	5.76 (2.61 - 12.7)	2.37 (1.28)
	Immune-Mediated Pancreatitis	3	11.86 (3.3 - 42.58)	3.25 (1.62)
General Disorders And Administration Site Conditions	Pyrexia	19	1.86 (1.17 - 2.95)	0.86 (0.19)
	Chest Discomfort	9	5.46 (2.72 - 10.95)	2.3 (1.33)
	Chills	7	5.26 (2.4 - 11.56)	2.26 (1.17)
	Temperature Intolerance	4	24.88 (7.27 - 85.11)	4.01 (2.47)
Hepatobiliary Disorders	Hepatic Function Abnormal	28	6.75 (4.52 - 10.09)	2.55 (1.97)
	Liver Injury	13	25.94 (13.03 - 51.62)	4.04 (3.13)
	Drug-Induced Liver Injury	5	3.82 (1.53 - 9.55)	1.84 (0.6)
	Autoimmune Hepatitis	4	5.44 (1.92 - 15.41)	2.3 (0.92)
	Immune-Mediated Hepatic Disorder	4	2.95 (1.07 - 8.13)	1.49 (0.15)
Immune System Disorders	Hypersensitivity	11	6.69 (3.53 - 12.65)	2.56 (1.66)
	Anaphylactic Shock	5	6.22 (2.43 - 15.91)	2.47 (1.21)
	Anaphylactoid Reaction	3	21.75 (5.43 - 87.08)	3.89 (2.18)
Investigations	White Blood Cell Count Decreased	65	17.91 (13.35 - 24.01)	3.64 (3.23)
	Neutrophil Count Decreased	60	18.32 (13.49 - 24.87)	3.66 (3.24)
	Granulocyte Count Decreased	17	57.62 (27.92 - 118.94)	4.65 (3.79)
	Platelet Count Decreased	14	3.05 (1.77 - 5.26)	1.53 (0.76)
	Haemoglobin Decreased	8	2.91 (1.42 - 5.96)	1.47 (0.47)
	Hepatic Enzyme Increased	6	5.94 (2.53 - 13.98)	2.41 (1.25)
	Blood Pressure Decreased	4	4.05 (1.45 - 11.29)	1.92 (0.56)
	Myocardial Necrosis Marker Increased	3	18.64 (4.81 - 72.19)	3.73 (2.05)

Metabolism And Nutrition Disorders	Decreased Appetite	18	1.83 (1.13 - 2.94)	0.83 (0.15)
Nervous System Disorders	Hypoaesthesia	7	3.68 (1.7 - 7.97)	1.79 (0.72)
Respiratory, Thoracic And Mediastinal Disorders	Immune-Mediated Lung Disease	6	2.64 (1.15 - 6.03)	1.34 (0.21)
	Dysphonia	4	3.05 (1.11 - 8.43)	1.54 (0.2)
	Tachypnoea	3	10.04 (2.86 - 35.27)	3.06 (1.45)
Skin And Subcutaneous Tissue Disorders	Rash	35	2.93 (2.07 - 4.14)	1.46 (0.96)
	Pruritus	32	5.15 (3.56 - 7.47)	2.21 (1.67)
	Drug Eruption	8	8.52 (3.98 - 18.22)	2.86 (1.81)
	Erythema Multiforme	6	11.89 (4.81 - 29.39)	3.25 (2.03)
	Rash Erythematous	4	9.67 (3.27 - 28.63)	3.01 (1.59)
	Dermatitis Exfoliative	3	18.64 (4.81 - 72.19)	3.73 (2.05)
Vascular Disorders	Flushing	4	4.58 (1.63 - 12.85)	2.08 (0.71)

Abbreviation: ROR, reporting odds ratio; IC025, the lower limit of the 95% CI of the IC; CI, confidence interval; PT, preferred term.

Supplementary Table 4 Signal strength of tislelizumab in patients in the Esophageal Neoplasms group from FAERS data

SOC	PT(Preferred Term)	a	ROR(95%Cl)	IC(IC025)
Blood And Lymphatic System Disorders	Myelosuppression	94	23.22 (16.52 - 32.63)	2.92 (2.52)
	Agranulocytosis	3	18.13 (3.02 - 108.92)	2.96 (1.09)
Hepatobiliary Disorders	Liver Injury	5	15.2 (4.06 - 56.88)	2.85 (1.35)
	Hepatic Function Abnormal	5	5.06 (1.77 - 14.44)	1.93 (0.57)
	Drug-Induced Liver Injury	5	20.27 (4.82 - 85.2)	3.02 (1.49)
Investigations	Neutrophil Count Decreased	21	7.01 (4.06 - 12.1)	2.21 (1.48)
	White Blood Cell Count Decreased	20	7.23 (4.12 - 12.68)	2.24 (1.49)
	Platelet Count Decreased	11	6.17 (2.96 - 12.83)	2.11 (1.13)
	Haemoglobin Decreased	4	48.52 (5.41 - 435.42)	3.38 (1.62)
	Granulocyte Count Decreased	4	12.12 (3.02 - 48.69)	2.7 (1.08)
Skin And Subcutaneous Tissue Disorders	Rash	7	4.06 (1.71 - 9.62)	1.7 (0.54)
	Pruritus	5	3.56 (1.31 - 9.72)	1.56 (0.23)
	Drug Eruption	4	24.25 (4.43 - 132.93)	3.11 (1.42)

Abbreviation: ROR, reporting odds ratio; IC025, the lower limit of the 95% CI of the IC; CI, confidence interval; PT, preferred term.

Supplementary Table 5 Signal strength of tislelizumab in patients in the Liver Neoplasms group from FAERS data

SOC	PT(Preferred Term)	a	ROR(95%Cl)	IC(IC025)
Blood And Lymphatic System Disorders	Myelosuppression	65	22.94 (14.49 - 36.32)	2.63 (2.15)
Endocrine Disorders	Hypothyroidism	3	6.04 (1.34 - 27.12)	1.94 (0.17)
Gastrointestinal Disorders	Mouth Ulceration	4	6.46 (1.72 - 24.2)	2 (0.41)
General Disorders And Administration Site Conditions	Chest Discomfort	4	4.61 (1.34 - 15.85)	1.71 (0.16)
Hepatobiliary Disorders	Hepatic Function Abnormal	11	5.33 (2.47 - 11.5)	1.82 (0.82)
	Liver Injury	4	32.36 (3.6 - 290.56)	2.84 (1.09)
Investigations	Neutrophil Count Decreased	6	12.21 (3.42 - 43.52)	2.43 (1.03)
Skin And Subcutaneous Tissue Disorders	Rash	7	4.38 (1.73 - 11.07)	1.65 (0.45)
	Pruritus	7	4.75 (1.85 - 12.16)	1.73 (0.51)
	Drug Eruption	7	5.18 (1.99 - 13.48)	1.8 (0.58)
	Erythema Multiforme	4	16.17 (2.95 - 88.7)	2.58 (0.89)

Abbreviation: ROR, reporting odds ratio; IC025, the lower limit of the 95% CI of the IC; CI, confidence interval; PT, preferred term.

Supplementary Table 6 Signal strength of tislelizumab in patients in the Nasopharyngeal Neoplasms group from FAERS data

SOC	PT (Preferred Term)	a	ROR(95%Cl)	IC(IC025)
Blood And Lymphatic System Disorders	Myelosuppression	54	2.09 (1.4 - 3.11)	0.54 (0.07)
Investigations	Neutrophil Count Decreased	24	4.65 (2.35 - 9.2)	1.13 (0.38)
Skin And Subcutaneous Tissue Disorders	Rash	9	7.67 (2.06 - 28.65)	1.37 (0.16)
	Pruritus	9	7.67 (2.06 - 28.65)	1.37 (0.16)

Abbreviation: ROR, reporting odds ratio; IC025, the lower limit of the 95% CI of the IC; CI, confidence interval; PT, preferred term.

Supplementary Table 7 Signal strength of tislelizumab in patients in the Stomach Neoplasms group from FAERS data

SOC	PT(Preferred Term)	a	ROR(95%Cl)	IC(IC025)
Blood And Lymphatic System Disorders	Myelosuppression	35	27.21 (17.15 - 43.17)	3.72 (3.11)
Investigations	Neutrophil Count Decreased	8	12.68 (5.65 - 28.46)	3.24 (2.14)
	White Blood Cell Count Decreased	7	11.8 (5.02 - 27.76)	3.17 (2.01)
Skin And Subcutaneous Tissue Disorders	Pruritus	5	11.17 (4.11 - 30.37)	3.13 (1.79)
	Rash	4	8.43 (2.84 - 24.99)	2.81 (1.37)

Abbreviation: ROR, reporting odds ratio; IC025, the lower limit of the 95% CI of the IC; CI, confidence interval; PT, preferred term.

Supplementary Table 8 Signal detection for ICI-associated irAEs.

ICI regimens	ROR (95% CI)
Monotherapy	
Anti-PD-1	
Tislelizumab	1.69 (1.56-1.83)
Nivolumab	2.21 (2.17-2.26)
Pembrolizumab	2.35 (2.28-2.43)
Cemiplimab	2.42 (1.94-3.01)
Anti-PD-L1	
Atezolizumab	2.27 (2.15-2.40)
Durvalumab	3.84 (3.53-4.19)
Anti-CTLA-4	
Ipilimumab	3.01 (2.89-3.14)
Tremelimumab	4.52 (0.76-27.04)
Combination therapy	
Ipilimumab + nivolumab	4.80 (4.41-5.23)
Ipilimumab + pembrolizumab	7.77 (5.34-11.30)
Durvalumab + tremelimumab	7.03 (1.82-27.18)

Note: The ROR value for Tislelizumab comes from this study, while data for other immune checkpoint inhibitors are referenced from the study by Chen et al. ICI: immune checkpoint inhibitor; ROR, reporting odds ratio; CI, confidence interval;

Supplementary Table 9 irAE signals associated with tislelizumab.

irAEs	N	ROR(95%Cl)	IC(IC025)
All*	726	1.69 (1.56 - 1.83)	0.64 (0.52)
Skin Toxicities*	278	2.61 (2.31 - 2.95)	1.31 (1.14)
Hepatitis*	159	4.36 (3.72 - 5.12)	2.07 (1.84)
Gastrointestinal toxicities	61	0.56 (0.43 - 0.72)	-0.82 (-1.19)
Endocrine Toxicities*	47	5.01 (3.76 - 6.68)	2.31 (1.89)
Cardiovascular Toxicities	47	0.86 (0.65 - 1.15)	-0.21 (-0.63)
Hematologic Toxicities*	45	1.54 (1.15 - 2.06)	0.62 (0.19)
Lung Toxicities*	39	4.6 (3.36 - 6.31)	2.19 (1.73)
Renal Toxicities	22	1.37 (0.9 - 2.08)	0.45 (-0.15)
Musculoskeletal Toxicities	17	0.23 (0.14 - 0.36)	-2.13 (-2.81)
Nervous System Toxicities	8	0.54 (0.27 - 1.09)	-0.88 (-1.84)
Ocular Toxicities	3	0.59 (0.19 - 1.82)	-0.77 (-2.21)

Abbreviation: Asterisks (*) indicate statistically significant signals in algorithm; ROR, reporting odds ratio; IC, information component; IC025, the lower limit of the 95% CI of the IC; CI, confidence interval; irAEs, immune-related adverse events.

Supplementary Table 10 Top 50 most frequent adverse events for tislelizumab excluding common medication co-usage at the PT level from FAERS data

SOC	PT(Preferred Term)	a	ROR(95%Cl)	IC(IC025)
Blood And Lymphatic System Disorders	Myelosuppression	538	184.84 (167.67 - 203.76)	7.1 (6.96)
	Anaemia	21	2.95 (1.92 - 4.53)	1.55 (0.93)
	Thrombocytopenia	21	4.39 (2.86 - 6.75)	2.12 (1.5)
	Leukopenia	16	7.21 (4.41 - 11.81)	2.84 (2.14)
	Agranulocytosis	7	7.77 (3.7 - 16.35)	2.95 (1.93)
Cardiac Disorders	Myocardial Injury	13	146.51 (83.19 - 258)	7.08 (6.28)
	Myocarditis	10	15.07 (8.08 - 28.12)	3.9 (3.02)
	Palpitations	10	2.48 (1.33 - 4.62)	1.31

				(0.43)
	Atrial Fibrillation	8	2.25 (1.13 - 4.51)	1.17 (0.2)
	Arrhythmia	4	2.87 (1.08 - 7.67)	1.52 (0.23)
	Immune-Mediated Myocarditis	4	20.2 (7.53 - 54.14)	4.32 (3.02)
	Cardiotoxicity	3	6.53 (2.1 - 20.3)	2.7 (1.25)
Endocrine Disorders	Hypothyroidism	16	9.89 (6.04 - 16.19)	3.29 (2.59)
	Adrenal Insufficiency	3	3.61 (1.16 - 11.21)	1.85 (0.4)
	Secondary Adrenocortical Insufficiency	3	23.85 (7.63 - 74.54)	4.56 (3.1)
Gastrointestinal Disorders	Gastrointestinal Disorder	19	3.29 (2.09 - 5.16)	1.71 (1.06)
	Mouth Ulceration	18	18.54 (11.64 - 29.54)	4.19 (3.52)
	Abdominal Distension	11	2.44 (1.35 - 4.42)	1.28 (0.45)
	Gastrointestinal Haemorrhage	7	4.05 (1.93 - 8.5)	2.01 (0.99)
	Immune-Mediated Pancreatitis	5	100.76 (40.93 - 248.04)	6.58 (5.36)
General Disorders And Administration Site Conditions	Pyrexia	46	3.09 (2.31 - 4.14)	1.61 (1.19)
	Asthenia	30	1.92 (1.34 - 2.75)	0.93 (0.41)
	Chest Discomfort	26	6.04 (4.1 - 8.89)	2.58 (2.02)
	Chills	11	2.4 (1.33 - 4.34)	1.26 (0.42)
	Temperature Intolerance	6	11.67 (5.23 - 26.08)	3.53 (2.44)
	Hyperpyrexia	5	23.83 (9.85 - 57.62)	4.55 (3.37)
Hepatobiliary Disorders	Hepatic Function Abnormal	83	50.98 (40.86 - 63.61)	5.59 (5.27)
	Liver Injury	42	24.79 (18.24 - 33.69)	4.59 (4.15)
	Drug-Induced Liver Injury	16	8.16 (4.98 - 13.35)	3.01 (2.31)

	Immune-Mediated Hepatic Disorder	8	24.91 (12.39 - 50.1)	4.62 (3.65)
	Autoimmune Hepatitis	7	26.34 (12.48 - 55.6)	4.7 (3.67)
	Hepatic Failure	7	8.28 (3.94 - 17.41)	3.04 (2.02)
	Jaundice	3	4.43 (1.43 - 13.77)	2.14 (0.7)
	Acute Hepatic Failure	3	6.38 (2.05 - 19.83)	2.67 (1.22)
Immune System Disorders	Hypersensitivity	26	3.59 (2.44 - 5.29)	1.83 (1.28)
	Anaphylactic Shock	8	7.41 (3.7 - 14.85)	2.88 (1.91)
	Anaphylactoid Reaction	5	39.61 (16.32 - 96.14)	5.28 (4.08)
Investigations	Neutrophil Count Decreased	109	49.48 (40.76 - 60.08)	5.53 (5.25)
	White Blood Cell Count Decreased	107	22.1 (18.2 - 26.85)	4.39 (4.11)
	Platelet Count Decreased	43	9.08 (6.71 - 12.28)	3.16 (2.72)
	Granulocyte Count Decreased	22	124.01 (80.41 - 191.24)	6.85 (6.23)
	Transaminases Increased	14	18.94 (11.17 - 32.1)	4.22 (3.47)
	Hepatic Enzyme Increased	13	3.69 (2.14 - 6.37)	1.88 (1.1)
	Oxygen Saturation Decreased	11	3.69 (2.04 - 6.67)	1.88 (1.04)
	Haemoglobin Decreased	10	2.52 (1.35 - 4.69)	1.33 (0.46)
	Heart Rate Increased	10	2.44 (1.31 - 4.55)	1.28 (0.41)
	Blood Pressure Decreased	9	3.42 (1.78 - 6.59)	1.77 (0.86)
	Cortisol Decreased	8	86.83 (42.69 - 176.62)	6.37 (5.38)
	Myocardial Necrosis Marker Increased	6	59.19 (26.23 - 133.55)	5.84 (4.73)
	Red Blood Cell Count Decreased	6	4.48 (2.01 - 9.98)	2.16 (1.06)
	Blood Thyroid Stimulating Hormone Increased	6	17.89 (8 - 40.01)	4.14 (3.05)

	White Blood Cell Count Increased	4	2.89 (1.08 - 7.7)	1.53 (0.23)
	Troponin I Increased	3	53.44 (16.95 - 168.51)	5.7 (4.23)
	Blood Creatine Phosphokinase Increased	3	3.51 (1.13 - 10.91)	1.81 (0.36)
	Blood Bilirubin Increased	3	3.61 (1.16 - 11.21)	1.85 (0.4)
	Breath Sounds Abnormal	3	10.49 (3.37 - 32.64)	3.38 (1.93)
	Gamma-Glutamyltransferase Increased	3	4.63 (1.49 - 14.37)	2.21 (0.76)
	Blood Lactate Dehydrogenase Increased	3	6.57 (2.11 - 20.42)	2.71 (1.26)
Metabolism And Nutrition Disorders	Decreased Appetite	33	3.05 (2.17 - 4.31)	1.6 (1.1)
	Hypokalaemia	7	3.6 (1.72 - 7.57)	1.84 (0.82)
	Diabetes Mellitus	6	2.41 (1.08 - 5.37)	1.27 (0.17)
	Hyponatraemia	6	3.38 (1.51 - 7.53)	1.75 (0.66)
	Diabetic Ketoacidosis	5	6.29 (2.61 - 15.14)	2.65 (1.46)
	Hypomagnesaemia	3	4.6 (1.48 - 14.28)	2.2 (0.75)
	Hypoproteinaemia	3	22.87 (7.32 - 71.46)	4.5 (3.04)
Nervous System Disorders	Hypoaesthesia	14	2.31 (1.37 - 3.91)	1.2 (0.45)
	Neurotoxicity	4	4.89 (1.83 - 13.06)	2.29 (0.99)
	Immune-Mediated Myasthenia Gravis	3	55.03 (17.44 - 173.6)	5.74 (4.27)
Psychiatric Disorders	Listless	4	35.64 (13.24 - 95.92)	5.13 (3.82)
Renal And Urinary Disorders	Renal Cyst	3	10.94 (3.51 - 34.05)	3.44 (1.99)
	Proteinuria	3	3.16 (1.02 - 9.83)	1.66 (0.21)
	Immune-Mediated Cystitis	3	347.37 (101.16 - 1192.86)	8.19 (6.61)
Respiratory, Thoracic And Mediastinal Disorders	Interstitial Lung Disease	27	12.3 (8.41 - 17.99)	3.6 (3.05)

	Immune-Mediated Lung Disease	8	38.17 (18.93 - 76.95)	5.22 (4.25)
	Dysphonia	8	3.03 (1.51 - 6.08)	1.6 (0.63)
	Tachypnoea	7	12.1 (5.75 - 25.47)	3.59 (2.56)
	Pneumonitis	5	3.61 (1.5 - 8.69)	1.85 (0.67)
Skin And Subcutaneous Tissue Disorders	Pruritus	98	4.86 (3.97 - 5.95)	2.24 (1.94)
	Rash	88	4.69 (3.79 - 5.8)	2.19 (1.88)
	Drug Eruption	31	40.69 (28.45 - 58.19)	5.3 (4.78)
	Erythema Multiforme	14	44.82 (26.34 - 76.26)	5.45 (4.69)
	Skin Exfoliation	9	2.17 (1.12 - 4.17)	1.11 (0.2)
	Rash Erythematous	7	2.92 (1.39 - 6.13)	1.54 (0.52)
	Palmar-Plantar Erythrodysaesthesia Syndrome	6	6.49 (2.91 - 14.48)	2.69 (1.6)
	Dermatitis Exfoliative	5	87.45 (35.63 - 214.62)	6.38 (5.17)
	Papule	5	14.64 (6.07 - 35.33)	3.86 (2.67)
	Rash Macular	5	3.14 (1.3 - 7.55)	1.65 (0.47)
	Dermatitis Bullous	4	16.55 (6.18 - 44.31)	4.03 (2.74)
	Skin Erosion	4	24.46 (9.12 - 65.66)	4.59 (3.29)
	Immune-Mediated Dermatitis	3	25.26 (8.08 - 78.98)	4.64 (3.18)
	Macule	3	26.47 (8.46 - 82.77)	4.7 (3.25)
	Pain Of Skin	3	3.54 (1.14 - 10.98)	1.82 (0.37)
Vascular Disorders	Flushing	9	3.57 (1.86 - 6.88)	1.83 (0.92)
	Cyanosis	3	5.98 (1.92 - 18.57)	2.57 (1.13)

Abbreviation: ROR, reporting odds ratio; IC025, the lower limit of the 95% CI of the IC; CI, confidence interval; PT, preferred term.
